# Anesthetic Management of an Obstetric Patient With Ehlers-Danlos and Wolff-Parkinson-White Syndromes

**DOI:** 10.7759/cureus.45486

**Published:** 2023-09-18

**Authors:** Sara K Biladeau, Braden Bocard, Ryan Grell

**Affiliations:** 1 Anesthesiology and Perioperative Medicine, University of Louisville School of Medicine, Louisville, USA

**Keywords:** wolff-parkinson-white syndrome, point-of-care ultrasound (pocus), cardiac anesthesia, cardiac arrythmia, epidural anaesthesia, ehlers danlos syndrome, obstetric anesthesia

## Abstract

A 31-year-old, primigravida, nullipara (G1P0) female with a past medical history of Ehlers-Danlos Syndrome (EDS), newly diagnosed Wolff-Parkinson-White Syndrome (WPW), and fetal breech presentation initially presented at 36+5 weeks gestation for an external cephalic version (ECV). The patient noted significant symptomatology related to her WPW which had worsened over the course of her pregnancy despite being started on oral metoprolol. Despite joint recommendations from the anesthesia and obstetric teams to combine the ECV with a same-day scheduled induction of labor or cesarean section, the patient declined. An epidural catheter was placed using ultrasound guidance and slowly titrated with 2% lidocaine; however, the ECV was unsuccessful. At 39 weeks gestation, the patient underwent an uncomplicated low transverse cesarean section under combined spinal-epidural anesthesia. The patient was discharged two days later in stable condition with a referral to an electrophysiologist. Here we describe the anesthetic preparation and management for an external cephalic version and subsequent cesarean section in a patient with these two rare conditions.

## Introduction

Rare genetic diseases provide a heightened level of complexity during the peripartum period [1-4]. Multiple rare diseases in a single parturient often pose an exceptional challenge. Wolff-Parkinson-White Syndrome (WPW) is a disease of abnormal cardiac conduction which, if left untreated, can lead to life-threatening arrhythmias [5]. A variety of medications can decrease the chances of a patient developing an arrhythmia during the stress of anesthesia; however, prompt action must be taken to correct it should one occur. Ehlers-Danlos Syndrome (EDS) is a disease of connective tissue and can affect multiple organ systems, including skin, uterus, and vasculature [6-7]. Appropriate discussion to ascertain which specific type of EDS a patient has enables anesthesiologists and obstetricians to better understand the possible complications during childbirth. Both WPW and EDS have specific anesthetic considerations to allow safe management of the parturient [1,6].

This article adheres to the applicable Enhancing the QUAlity and Transparency Of health Research (EQUATOR) guideline. Written Health Insurance Portability and Accountability Acts (HIPAA) authorization was obtained from the patient.

## Case presentation

A 31-year-old, primigravida, nullipara (G1P0) female with a past medical history of myopathic EDS, newly-diagnosed WPW, and fetal breech presentation initially presented to us at 36+5 weeks gestation for an external cephalic version (ECV). Her EDS symptoms were notable for frequent dislocations of her joints without any long-term sequelae or known spinal abnormalities. The patient reported a lifelong history of palpitations, dizziness, and fainting, initially misdiagnosed as postural orthostatic tachycardia syndrome. During a peripartum cardiology consultation for EDS evaluation of the aorta, an electrocardiogram (EKG) was ordered, and she was formally diagnosed with WPW with inducible orthodromic atrioventricular reentrant tachycardia (AVRT). At that time, she was started on 25mg oral metoprolol daily and scheduled for follow-up to consider a post-delivery electrophysiological ablation of the cardiac accessory conduction pathway(s).

**Figure 1:**
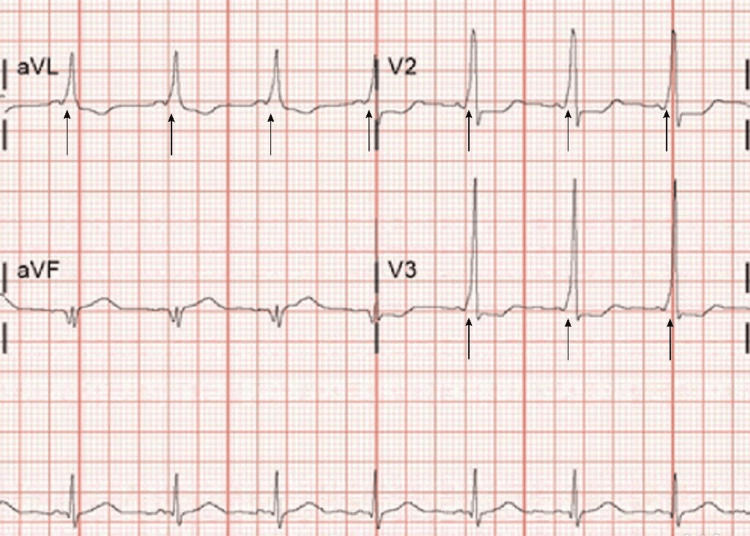
Patient's 12-lead EKG Demonstrating Delta Waves Consistent with WPW EKG: Electrocardiogram; WPW: Wolff-Parkinson-White Syndrome

The patient was seen in the anesthesia preoperative clinic where she described a mild reduction of WPW symptoms since her cardiology visit. A focused physical examination was performed and revealed no worrisome cardiac findings. Her spine examination revealed no scoliosis and normal-appearing intervertebral spaces. Her CBC revealed a hemoglobin of 11.2 g/dL, consistent with normal physiologic anemia of pregnancy, and a platelet count of 144x10^3^/uL. Her EKG demonstrated the characteristic delta waves with a heart rate in the 80s (Figure 1). The anesthesia, obstetric, and cardiology teams jointly recommended combining the ECV with a same-day scheduled induction of labor (IOL) or cesarean section to minimize the chances of EDS-related vascular injury during the placement of an epidural and to prevent significant adrenergic surges that might induce tachyarrhythmias, though the patient initially declined elective IOL or operative intervention. 

On the day of her ECV at 37 weeks gestation, the patient was brought into the operating room and connected to the American Society of Anesthesiologists (ASA) standard monitors. A variety of cardiac medications including adenosine, metoprolol, and phenylephrine and a code cart with a defibrillator were also brought to the room. She received a 500 mL bolus of crystalloids to minimize the risk of hypotension. An epidural catheter was placed at the L3/4 interspace in a single attempt using ultrasound guidance to minimize hematoma risk. After a 3 mL test dose of standard 1.5% lidocaine with epinephrine 1:200,000, her epidural was dosed with 3 mL of 2% lidocaine every 3-5 minutes until the T6 dermatome was insensitive to cold and pinprick testing. The total volume of lidocaine administered was 12 mL. Once the appropriate analgesic level was obtained, the obstetricians attempted the ECV; however, they were unsuccessful after multiple attempts. The procedure was aborted, and the patient was brought to the post-anesthesia care unit in stable condition.

After a thorough discussion between both teams and the patient, the patient chose to schedule an elective cesarean section at 39 weeks gestation. Her epidural was removed, and she was observed for one hour. She was then discharged with strict instructions to immediately present to the nearest hospital if she should have changes in cardiac or neurologic status.

At 39 weeks gestation, the patient presented for her scheduled cesarean section. All cardiac emergency medications and a defibrillator were readily available. The patient was brought into the operating room where standard ASA monitors were placed, and a bolus of 500 mL of crystalloids was administered. A combined spinal-epidural was placed in the L3/L4 interspace using an intrathecal dose of 10.5 mg of 0.75% bupivacaine in 8.25% dextrose in the usual fashion. She was placed supine with left uterine displacement, and phenylephrine infusion at 0.2 mcg/kg/min was initiated to maintain normotension. She was noted to be insensitive to cold and pinprick testing at the T4 dermatome. The surgeons sterilely prepped and draped the patient in the usual fashion and performed an Allis clamp test which was negative in all four abdominal quadrants. 

The cesarean section was completed in approximately 70 minutes without any surgical or cardiac complications. The patient tolerated the procedure very well. Blood loss was approximately 300 mL. The uterine tone was excellent throughout and only required a prophylactic oxytocin infusion to prevent post-delivery uterine atony. Near the conclusion of the case, her epidural was slowly bolused with 10 mL of 0.25% bupivacaine and 50 mcg of fentanyl to improve post-operative analgesia. Her epidural catheter was then removed, and a sterile dressing was applied. Her vital signs remained stable throughout her intra- and post-operative course and had appropriate resolution of her motor blockade within one hour of the conclusion of the procedure. She was monitored in a telemetry unit overnight.

On post-operative day one, she demonstrated a full return of neurologic status with no back pain, no signs of hematoma at her combined spinal and epidural anesthesia (CSE) site, and no symptoms of palpitations or pre-syncope. The patient was safely discharged home two days later in stable condition with a referral for an electrophysiologist.

## Discussion

WPW syndrome is a rare cardiac conduction disorder where, in addition to the normal atrioventricular conduction system, an accessory pathway exists. There are two characteristic patterns seen on EKG, a shortened PR interval (<0.12 seconds) due to the quicker conduction speed down the atrial accessory pathway compared to the normal pathway as well as a delta wave (upsloping of the QRS wave) with a widened QRS seen with pre-excitation during ventricular depolarization. The accessory pathway allows for AVRT to occur in two distinct patterns: the more common orthodromic (the electrical signal travels normally through the AV node to the ventricles then travels retrograde to the atria) or the less common antidromic (the electrical signal travels down the atria to the ventricles through the accessory pathway then retrograde through the AV nodal tissue) [1].

The incidence of supraventricular arrhythmias in patients with WPW is increased during pregnancy as there is increased sensitivity to adrenergic stimuli such as stress, anxiety, or estrogen. Estrogen is thought to increase the excitability of uterine muscle fibers, the number and sensitivity of alpha-adrenergic receptors, and the adrenergic receptors in the hypothalamus. This combination can lead to arrhythmias by changing the refractory period or conduction velocity in the reentrant circuit [2].

Although general and neuraxial anesthesia can safely be used, both can increase the potential for arrhythmias [1]. To minimize risk, it is important to maintain a balance between sympathetic and vagal tone as both extremes can lead to arrhythmias. Neuraxial anesthesia is particularly beneficial as it can prevent the propagation of pain during labor or cesarean sections; however, it can cause significant hypotension. Typically, this can be prevented or managed with alpha-adrenergic agonists. Neuraxial anesthesia also avoids laryngoscopy and neuromuscular blockade reversal agents like neostigmine, both of which can increase sympathetic stimulation. In a situation where general anesthesia is necessary, various preventative measures can be taken to reduce the chances of inducing AVRT (Table 1). Regardless of technique, adequate intra- and post-operative pain control should be emphasized as pain can trigger life-threatening arrhythmias [3].

**Table 1:** Anesthetic Considerations to Prevent AVRT in Patients With WPW

Preferred Interventions	Interventions That Can Trigger AVRT
Fluid bolus (maintains preload, prevents sympathetic response to hypotension)	Ephedrine, epinephrine, ketamine (sympathomimetic)
Phenylephrine (vasopressor of choice)	Halothane (pro-arrhythmic)
	Meperidine, metoclopramide, and pancuronium (induce tachycardia)
	Atracurium (histamine release)
	Succinylcholine (increases vagal tone)

Even with a carefully crafted anesthetic plan, dysrhythmia may still occur; and anesthesiologists should be prepared to intervene immediately when necessary. Current guidelines for the management of adult patients with supraventricular arrhythmias focus on terminating AVRT rather than blocking the AV node as the latter can lead to life-threatening ventricular fibrillation (Table 2) [4-5]. It is imperative, at minimum, to have the medications described as well as a defibrillator immediately available as intervention must be prompt once AVRT is recognized [1].

**Table 2:** Acute Management of WPW ^a ^use if no ischemic/structural heart disease; ^b ^treatment if medications do not provide sufficient relief of symptoms AVRT: Atrioventricular reentrant tachycardia; WPW: Wolff-Parkinson-White Syndrome; IV: Intravenous

Prevention	Treatment: Hemodynamically Stable AVRT	Treatment: Hemodynamically Unstable AVRT
Class 1A drugs (procainamide, quinidine)^a^	Vagal maneuvers	Synchronized cardioversion
Class 1C drugs (propafenone, flecainide)^a^	Carotid massage	Defibrillation
Beta blockers	IV beta blockers	
Radiofrequency ablation^b^	IV adenosine	

EDS is a connective tissue disorder with a variety of genetic mutations leading to different subtypes (Table 3) [6]. Though our records support a diagnosis of hypermobile EDS by her cardiologist with significant joint dislocations endorsed by the patient, the patient had a COL1A1 mutation, seen in multiple different subtypes of EDS. It is important to note that, even though a patient will typically present with one subtype and its characteristic findings, the disease can affect multiple organ systems. There are no clear guidelines for anesthesia type or delivery method in patients with EDS [7]. Of note, regional anesthesia seems controversial as there is an increased risk of block failure seen with hypermobility EDS as well as hematoma formation seen with vascular EDS [8-10]. Nevertheless, multiple case reports have shown success with neuraxial anesthesia in parturient patients [9,11]. To the best of our knowledge, no case report of these two rare diseases presenting in a peripartum patient has been reported.

**Table 3:** EDS Subtypes, Clinical Features, Inheritance Pattern, Genetic Defect(s), and Anesthetic Considerations

Subtype	Clinicial Features	Inheritance Pattern	Genetic Defect	Anesthetic Considerations
Hypermobile	Generalized joint and skin hypermobility, musculoskeletal complications such as pain	Autosomal dominant	Unknown	Neuraxial less effective (recommend combined spinal-epidural)
Classic	Generalized joint and skin hypermobility and atrophic scarring	Autosomal dominant	Type V collagen (COL5A1, COL5A2); more rarely type I collagen (COL1A1)	High prevalence of aortic root dilation (echocardiography warranted)
Vascular	Arterial rupture, bowel perforation, uterine rupture, and carotid-cavernous sinus fistula	Autosomal dominant	Pro-alpha-1 chains of type III collagen (COL3A1); more rarely type I collagen (COL1A1)	Postpartum hemorrhage is more common, likely secondary to a higher likelihood of vascular and uterine rupture

## Conclusions

The anesthetic management for obstetric patients can be very complex, especially in patients with multiple comorbidities with potentially profound anesthetic implications such as WFW and EDS. Care must be taken to elucidate as accurate of a history and physical examination as possible to appropriately risk stratify and to inform anesthetic and obstetric management. A multidisciplinary discussion, including anesthesiologists, obstetricians, cardiologists, electrophysiologists, and maternal-fetal medicine specialists is warranted. Appropriate medication, equipment, and other resource availability must be confirmed to improve the likelihood of a safe patient outcome. Finally, appropriate post-operative referrals should be considered, particularly in patients with symptomatic WPW or in patients with aortic manifestations of EDS.
